# Spatiotemporal analysis of *Plasmodium falciparum* erythrocyte binding antigen-175 gene dimorphism in Ghana

**DOI:** 10.1186/s12936-025-05263-3

**Published:** 2025-01-22

**Authors:** Abraham Y. Kpirikai, Belinda A. Ofosu, Josie N. A. Okai, Victor Kornu, Abdul Rashid Kassim, Esther Donkor, Frederica Malm, Osumanu Ahmed, Mona-Liza E. Sakyi, Samirah Saiid, Albert Yao Kudakpo, Charles Mensah, Francis Dzabeng, Collins Morang’a, Gordon A. Awandare, Yaw Aniweh, Lucas N. Amenga-Etego

**Affiliations:** 1 West African Centre for Cell Biology of Infectious Pathogens, Accra University of Ghana, Volta Rd, Accra, Ghana; 2 Department of Biochemistry, Cell and Molecular Biology, University of Ghana, P. O. Box LG 54, Accra, Ghana

**Keywords:** Malaria, EBA-175 dimorphism, *Plasmodium falciparum*, Merozoites, Erythrocytes, Glycoprotein A (GpA), Alleles

## Abstract

**Background:**

Malaria remains a leading cause of death worldwide, claiming over 600,000 lives each year. Over 90% of these deaths, mostly among children under 5 years, occur in sub-Saharan Africa and are caused by *Plasmodium falciparum*. The merozoites stage of the parasite, crucial for asexual development invade erythrocytes through ligand-receptor interactions. Erythrocyte binding antigen (EBA)-175 is one of the key ligands facilitating invasion via interaction with glycoprotein A (GpA) receptors on the erythrocytes. EBA-175 is known to exist in two dimorphic allelic (F and C) forms with each found to infer different virulence. There is paucity of data on the prevalence of these alleles and their epidemiology in the Ghanaian malaria landscape and hence this study.

**Methods:**

Parasite gDNA was extracted from archived Dried Blood Spots (DBS) prepared from 700 confirmed malaria-infected individuals and analysed for *P. falciparum* EBA-175 dimorphism. Selective *eba-175* gene amplification via nested PCR and allele scoring using agarose gel electrophoresis for F, C and F/C alleles.

**Results:**

Of the total 632 successfully genotyped samples, prevalence of F, C, and F/C allelic forms were 61.2% (n = 387), 20.7% (n = 131), and 18.0% (n = 114), respectively. Seasonality analysis did not reveal a statistically significant difference in the prevalence of dimorphic forms between the wet (n = 475) and dry (n = 157) seasons (p = 0.051). The prevalence ratio (wet/dry) for C, F and F/C were determined to be 1.0, 1.1 and 1.4, respectively. Between 2019 and 2022, the prevalence of the alleles changed significantly (χ^2^ = 6.5427, p = 0.03). Geometric mean parasite density for the C, F, and F/C alleles were 21,477.1 [95%CI 15,749.2 − 29,288.1], 18,308.0 [95%CI 15,149.9–22,124.5] and 22,690.4[95% CI 16,891.9–30,479.2], respectively.

**Conclusion:**

The F-allele was the most prevalent form across all age groups, followed by the C allele and mixed F/C alleles. No significant difference in allele prevalence was observed between the high malaria season (wet) and low malaria season (dry). However, a statistically significant difference in the temporal prevalence of pure alleles (F & C) between two time points was observed.

The current study adds to the existing body of knowledge on *eba-175* allelic dimorphism and highlights the co-circulation of alleles in high malaria endemic areas in Ghana.

## Background

*Plasmodium falciparum* is responsible for the most severe form of malaria [1]. It causes fever, chills, and potentially life-threatening complications, such as cerebral malaria and multiorgan failures [2]. The parasite has a complex life cycle that involves both human and mosquito hosts with the merozoite stage being crucial for invasion of human red blood cells [3]. The process of merozoites invasion is aided by receptor-ligand interactions involving parasite proteins, such the 175-kDa erythrocyte binding antigen (EBA-175) expressed on the parasite's merozoite surface [4]. The binding of EBA-175 to erythrocytes is specific to glycophorin A (GpA), and requires the presence of N-acetylneuraminic acid (Neu5Ac), a sialic acid, on the host cell surface [5].

EBA-175 is a member of the Duffy-binding-like erythrocyte-binding protein (DBL-EBP) family, which include key adhesion molecules required for malaria parasites invasion [6]. The EBA-175/GpA pathway is the primary chymotrypsin-resistant invasion pathway [7]. Antibodies against EBA-175 can inhibit binding to GpA and block invasion of merozoites in vitro [8]. The protein is shown to have an N-terminal signal peptide, cysteine-rich DBL domains, a carboxyl cysteine-rich region (C-Cys), a transmembrane domain, and a short cytoplasmic tail [9, 10]. EBA-175 comprises of three domains: an N-terminal ligand binding domain (LBD), a central cysteine-rich domain (CRD), and a C-terminal transmembrane domain (TMD). The LBD binds to GpA on erythrocytes with high affinity, and the CRD helps to stabilize the binding [11]. This interaction triggers re-orientation of the merozoite surface, leading to the formation of a tight junction between the merozoite and erythrocyte surface [12]. Merozoites then secrete additional proteins, which aid in the entry process [3]. The role of EBA-175 in the invasion of erythrocytes is critical for the survival of the malaria parasite [13].

The *eba-175* gene is located on chromosome 7 and comprising four exons and with seven distinct regions [14]; region III is notably dimorphic, containing an insertion of 423 base-pair segment (F-allele) in Fajara, Gambia, strain 3 (FCR3) or a 342 base-pair segment (C-allele) in the (Malayan Camp strain) CAMP. Since *Plasmodium* merozoites are haploid, this dimorphism is conserved across strains, with only one of the two segments present in uniclonal infections [15–20]. The interaction of the dimorphic segments of the region III with the glycophorin backbone is thought to facilitate the smooth entry of the parasite through the erythrocyte membrane. Some studies report that this dimorphism, F- and C-alleles, infer different parasite virulence. C-allele variant has been found in absolute linkage disequilibrium with specific single nucleotide polymorphisms (SNPs). This could imply that the C-allele allele is favoured under specific environmental or immunological conditions, potentially impacting the virulence and adaptability of the parasite [15, 21, 22]. However, in a high malaria transmission region in Uganda, the F-allele was more prevalent in children under 5 years old compared to those over 10 years [23].

Since EBA-175 plays a crucial role in merozoite invasion of erythrocytes, it is a promising target and one of the key vaccine candidates being developed. However, the existence of dimorphic alleles (F and C) presents a significant challenge for vaccine design. Understanding the prevalence, distribution and potential functional differences between alleles is crucial for developing a broadly effective vaccine. If one allele confers a selective advantage or is associated with increased virulence, as some studies suggests, a vaccine targeting only one form might exert selective pressure favoring the other allele, potentially reducing vaccine efficacy over time. Therefore, elucidating the patterns of allelic distribution across different geographical regions, and age groups, such as this study aims to do in Ghana, can inform the development of vaccines that account for this genetic diversity.

Moreover, the association between *eba-175* alleles and factors such as parasite density, age, and transmission intensity could have significant implications for vaccine efficacy in different populations. If certain alleles are more prevent in specific age groups or ecological zones, this information could guide the design of targeted vaccination strategies. For instance, if F-allele is indeed more common in younger children, as observed in Uganda, vaccines might need to be tailored to age-related specific allelic prevalence. Also, understanding how allelic distribution relates to parasite density and clinical outcomes could help predict vaccine performance in different epidemiological settings. By providing comprehensive data on *eba-175* allelic dimorphism in Ghana, this study contributes valuable insights that can inform the development of more effective and broadly applicable malaria vaccines, potentially improving public health outcomes in endemic regions.

Therefore, understanding this nuanced distribution of the dimorphic alleles and their association with different eco-epidemiological parameters in Ghana will contribute to the understanding of EBA-175, and aid in therapeutic interventions that target the disruption of EBA-175 interaction.

This study aimed to assess the prevalence and distribution of the dimorphic alleles (F- and C-alleles) of the EBA-175 in malaria-infected individuals in Ghana. The focus was on the three malaria transmission areas in Ghana, identified as ecological zones: the Northern Savannah Zone, Middle Transitional/Forest Zone, and Coastal Savannah Zone [24]. Here, the prevalence of these alleles and their seasonal patterns were examined, while also investigating potential correlations between EBA-175 dimorphism and the parasite densities. Furthermore, how age groups might influence allelic variation were examined to determine whether there are notable differences in allele distribution among different age brackets, as well as gender.

## Methods

### Study area and subjects

This spatiotemporal study as part of a larger study was carried out with samples collected all year round in 2019 (n = 311) and 2022 (n = 321). These samples were collected from 32 sentinel health facilities located in fourteen of the sixteen administrative regions in Ghana. The samples collected were further grouped into three ecological zones: the Northern Savannah Zone, Middle Transitional/Forest Zone, and The Coastal Savannah Zone to reflect the malaria transmission pattern in Ghana.

Malaria transmission differs among the three ecological zones in Ghana due to varying climatic and environmental conditions that influence the propagation of *Anopheles* mosquitoes and malaria parasites transmission patterns. The Coastal Savannah and Forest zones exhibit a bimodal rainfall pattern, allowing for two peaks of malaria transmission, while the Northern Savannah zone experiences seasonal malaria transmission due to a unimodal rainfall pattern [25].

A total of 632 dried blood spots samples from malaria positive patients were collected from 32 different health centres across Ghana with their biodata well documented were successfully genotyped.

### Sample collection, processing, and DNA extraction

This study is part of a larger malaria molecular surveillance (MMS) study across 32 health facilities used by the Ghana National Malaria Elimination Programme (NMEP) as sentinel sites for drug efficacy studies. Out-patients reporting fever (or history of fever) within the past 24 h at these health facilities were screened with OnSite^®^ Malaria Rapid Diagnostic Test (mRDT) kits*.* Individual informed consent was documented for patients who tested positive and were willing to participate in the larger MMS studies. In accordance with the WHO guidelines [26–28], approximately 5 mL of whole blood was collected from consented participants into an EDTA tube, two 50 μL drops was spotted on a filter paper (Whatman No. 2) to make a dried blood spots (DBS) with 6 μL and 2 μL to prepare thick and thin malaria blood smears, respectively for parasite identification and quantification. *Plasmodium falciparum* density was determined by standard microscopy and asexual parasites were scored against 200 white blood cells (WBCs). Malaria smears were considered negative after examining 100 fields without detecting asexual or sexual parasites. The parasite density per 200 WBCs was converted to density per microlitre assuming 8000 WBCs.

The parasite DNA was extracted from DBS using the QIAamp^®^ DNA Investigator Kit (QIAGEN Catalogue No. 56504), following the manufacturer's instructions with minor modifications. The incubation time at 56 °C was extended to 17 h at 600rpm. The speed of the final centrifugation step at 14,000 rpm for 3 instead of 1 min to dry the membrane. These modifications were aimed at optimizing DNA yield and purity. The DNA concentration was determined using a QuBit Flex 4 fluorometer.

### Genotyping of *eba-175* alleles

To confirm genotypes of *P. falciparum*, nested PCR was conducted using a set of oligonucleotide primers as described by Touré et al*.* [17] with modifications of the annealing temperature to 57 °C for the first round and 60 °C for the second round. The primer concentrations remained unchanged, but the reaction volumes were altered to 11 µL. For the outer PCR, the following primers were used: forward primer (EBA-175 F1) 5′—ATTAACGCTGTACGTGTGTCTAG-3′, and reverse primer (EBA-175 R1) 5′—TCTCAACATTCATATTAACAATTC—3′. For the inner PCR, forward primer (EBA-175 F2) 5′—AAGAAATACTTCATCTAATAACG—3′, and reverse primer (EBA-175 R2) 5′—CAATTCCTCCAGACTGTTGAACAT-3'. The lyophilized primers were reconstituted to 100 µM.

The first round of nested PCR constituted a mixture of 0.25 µL (100 µM) of each forward and reverse primer, 5.5 µL of 2X Taq Plus master Mix II (cat: M0270L) buffer, 3 µL of nuclease-free water, and 2 µL of DNA template (1.2 to 1.5 ng/µL), giving a total PCR reaction volume of 11 µL. The second round of PCR used a similar mixture but with 4 µL of nuclease-free water and 1 µL of the amplicon from the first round. The cycling conditions for the first round included an initial denaturation at 96 ºC for 3 min, 30 cycles of denaturation at 96 ºC for 10 s, annealing at 57 ºC for 10 s, extension at 72 ºC for 50 s, followed by a final extension at 72  C for 10 min.

The second round of PCR had similar cycling conditions, with an initial denaturation at 95  C for 5 min, 30 cycles of denaturation at 95  C for 10 s, annealing at 60  C for 8 s, and extension at 72  C for 50 s. The final extension was also 72  C for 10 min.

The PCR products were resolved on 2% agarose gel electrophoresis, with 3.5 µL of the PCR product and 1.5 µL of loading dye (purple 6x) and a low weight molecular DNA marker (100 to 1500 bp, New England Biolabs) for allele analyses. The gel was run at 75 V for 90 min, and the band separations were visualized with an Amersham Imager 600. The weights of the alleles were used to categorize them into either a C or an F allele.

### Statistical analysis

Statistical analyses were conducted using R (version 4.4.0). The frequency of F and C alleles was determined for the entire sample set and by ecoepidemiological zones delineated as the Northern Savannah, the Middle Transitional/Forest belt, and the Coastal Savannah Zones.

To assess potential significant correlation between the alleles’ counts in different seasons, chi-squared analysis was used. The geometric mean parasite density (GMPD) was calculated with 95% CI in the different ecological zones, age groups, gender, and seasons. Kruskal–Wallis and one-way ANOVA tests were also employed to examine whether there was a significant correlation between parasitemia and the other parameters (sex, age, year). Further descriptive statistical measures were used to evaluate seasonal patterns of allele prevalence and explore possible relationships between the dimorphic alleles and parasitemia levels.

## Results

### Clinical and demographic characteristics of patients

A total of 632 samples were successfully genotyped for the different alleles of the eba-175 allele types at two time points, 311 in 2019 and 321 in 2022. Of the 632 patients, 554 had gender recorded, of whom, 46.8% (259/554) were males and 53.2% (295/554) were females. The mean age (completed years) was 20.8 years (SD =  ± 17.1). Overall, the geometric mean parasite density (GMPD) was 19,670.87 [95%CI 17,052.5–22,691.2] (Table 1).

**Table 1:** Prevalence of eba-175 alleles, mean ages, gender distribution and GMPD by eco-epidemiological zones

Transmission-zone	n	Prevalence (%)	Mean Age (yrs)	^a^GMPD [95% CI]	^b^Gender dist. (M/F)
Overall, n	**632**	**100**	**20.8**	**19,670.9 [17052.5–22,691.2]**	**554**
C-allele	131	20.7	22.0	21,477.1 [15749.2–29,288.1]	M: 55
					F: 62
F-allele	387	61.2	20.1	18,308.0 [15149.9–22,124.5]	M: 157
					F: 184
Mixed allele	114	18.0	21.8	22,690.4 [16891.9–30,479.2]	M: 47
					F: 49
Coastal Savannah	**302**	**100**	**23.7**	**20,838.5 [17260.6–25,158.1]**	**226**
C-allele	72	23.8	23.7	23,801 [16610.8–34,104.9]	M: 31
					F: 27
F-allele	168	55.6	23.7	19,520.2 [14990.6–25,418.5]	M: 67
					F: 56
Mixed allele	62	20.5	23.3	21,317.1 [14309.4–31,756.7]	M: 26
					F: 19
Middle transitional/forest	**162**	**100**	**20.1**	**14,884.6 [11083.8–19,989.0]**	**161**
C-allele	29	17.9	21.7	14,858.2 [7356.9–30,007.6]	M: 14
					F: 15
F-allele	112	69.1	19.9	13,663.8 [9517.1–19,617.4]	M: 49
					F: 62
Mixed allele	21	13.0	18.5	23,552.36 [11419.4–48,576.7]	M: 6
					F: 15
Northern Savannah	**168**	**100**	**16.4**	**23,204.1 [171.7–31,412.3]**	**167**
C-allele	30	17.9	18.1	23,963.0 [10767.9–53,327.7]	M: 10
					F: 20
F-allele	107	63.7	14.7	22,486.9 [15238.8–33,182.4]	M: 41
					F: 66
Mixed allele	31	18.4	20.8	25,066.7 [14222.3–44,180.0]	M: 15
					F: 15

M is male, and F is Female

^a^GMPD is the geometric mean parasite density and CI is the 95% confidence interval

^b^Gender distribution

The dominance of the F-allele was reflected across the local eco-epidemiological landscape with a higer prevalence in forest transitional zone, followed by Northern Savannah and Coastal zones (Table 1). A pattern that reflects the gradient of malaria transmission across these three zones

### Allele prevalence between eco-epidemiological zones

**Fig. 1:**
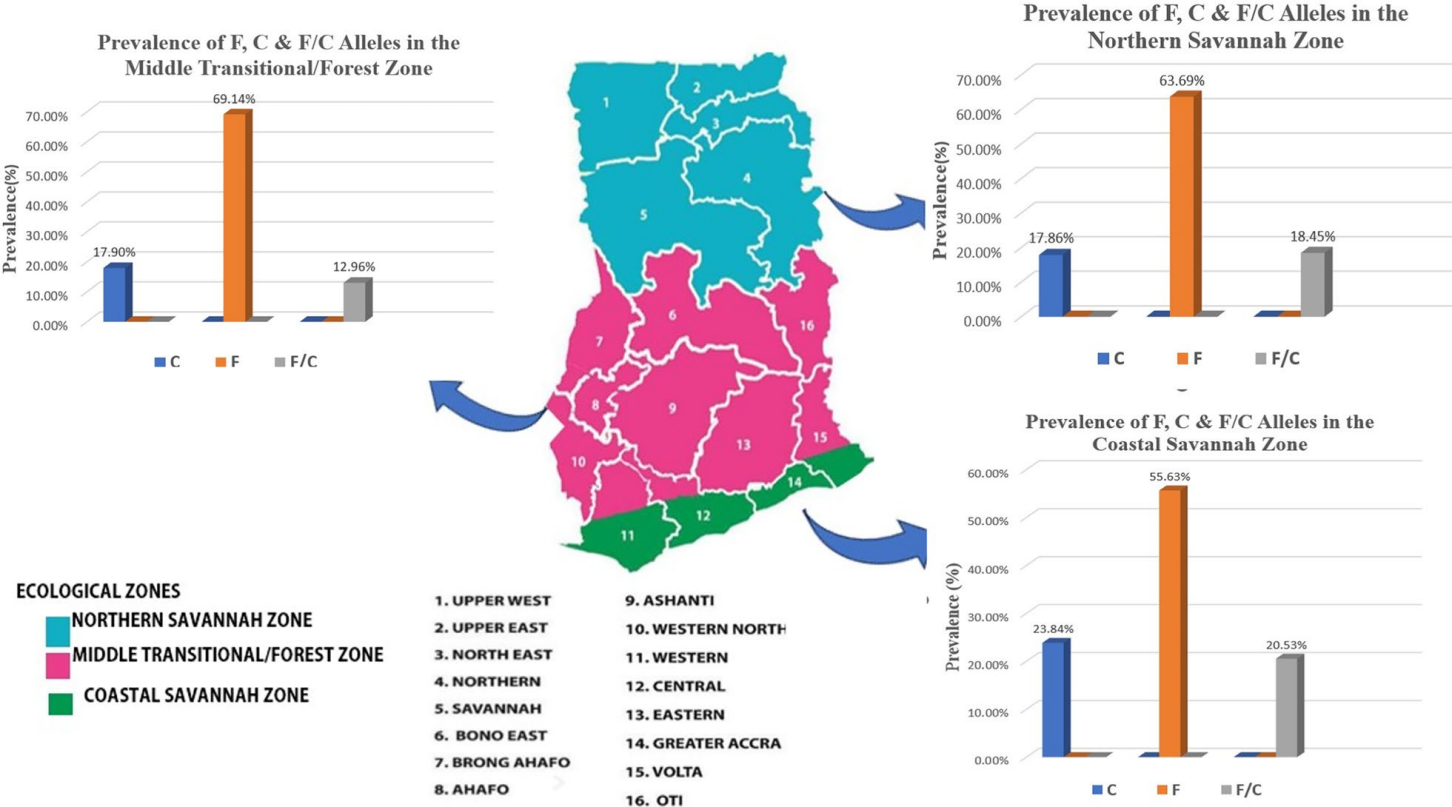
Distribution of *eba 175* gene allelic forms among the three eco-epidemiological transmission zones (color coded blue for the Northern Savannah zone, pink for the middle transitional/forest and green for coastal savannah zone), each with numbers that identifies the respective administrative regions that correspond with the key below figure. The prevalence of the different allelic forms (C, F and F/C) for each of the zone have been incorporated as insert

In the coastal ecological zone where malaria transmission intensity is relatively lower, the observed prevalence of the eba-175 alleles was 23.8% for the C-allele, 55.6% for the F-allele and 20.5% mixed infections (F/C-alleles). The GMPD for the coastal zone was 20,838.5 [95%CI 17,260.6–25,158.1]. The observed prevalence in the middle transitional/forest zone was 17.9% C-allele, 69.1% for F-allele and 13.0% mixed infection (F/C-alleles). The GMPD was 14,884.6 [95% CI 11,083.8–19,989.0]. In contrast, the prevalence in the northern Sahelian savannah zone was 17.9% for C-allele, 63.7% for F-allele and 18.4% for mixed infections (F/C-alleles). The GMPD was 23,204.1[95%CI 171.7—31,412.3]. Females showed higher prevalence of all alleles compared to males; mean age are also provided for each allele within these zones. Further details may be reviewed in Table 1 and Fig. 1.

### Allele prevalence by transmission season

The prevalence of the *eba-*175 alleles during the high malaria season (wet: May–October) was 19% (91/475) for the C-allele, 61% (290/475) for the F-allele, 20% (94/475) mixed infections with an overall GMPD of 1850.89 [95%CI 15,680.3–22018]. Prevalence in the low malaria season (dry: November–April) was 25% (40/157) for the C-allele, 62% (97/157) for the F-allele, 13% (20/157) for mixed infections with overall GMPD of 23,373.54.2 [95%CI 18,063.2–30,245.1]. However, seasonal differences in allele prevalence were not statistically significant (Chi-squared p = 0.06).

### Prevalence of eba-175 alleles by age group

**Fig. 2:**
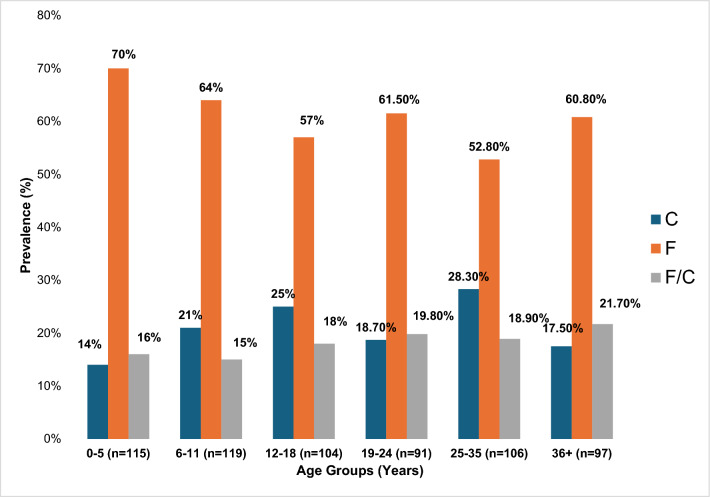
Distribution of the eba-175 allelic forms by age group. A bar chart showing the prevalence (%) of the C (Blue bars), F (orange bars) and F/C (grey bars) forms of eba-175 segregated according to the different age groups (0–5, 6–11, 12–18, 19–24, 25–35 and 36 +) in years. n = samples size

The bar chart illustrates the prevalence (%) of the F, C, and F/C alleles across different age groups. The F-allele consistently shows the highest prevalence across all groups, peaking at 70% in the 0–5 years age group and remaining above 50% in all other groups. The C-allele exhibits a varying pattern, with increased prevalence in older age groups, particularly 12–18 years (25%) and 25–35 years (28.3%). The combined F/C allele remains relatively stable, with the highest prevalence (21.7%) observed in the 36 + years group as shown in Fig. 2.

### Temporal prevalence of eba-175 alleles

A total of 49.2% (311/632) of the samples were collected in 2019 and the remaining 50.8% (321/632) were collected in 2022. Of the samples collected in 2019, the F-allele remained the most prevalent at 66.0% followed by C-allele and mixed C/F-allele at 17.0% each. On the one hand, in 2022 (n = 321) prevalence of the F-allele was 57.0% followed by the C-allele and mixed C/F-alleles was 24.0% and 19.0% respectively. The temporal difference in the occurrence of F, C and F/C alleles between the two time points, 2019 and 2022, was statistically significant (χ^2^ = 6.54, p = 0.03).

## Discussion

**Fig. 3:**
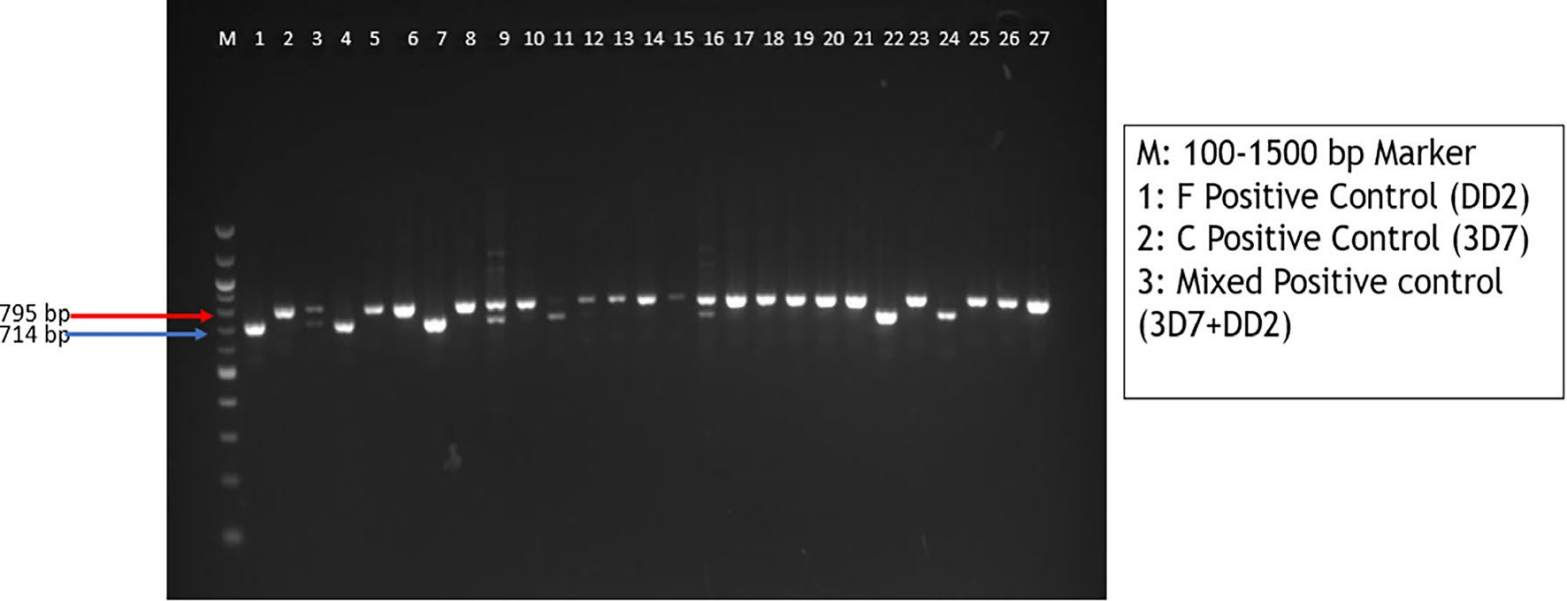
Representative agarose gel image of nested PCR products showing F and C alleles. Mixed allele is observed in lanes 3, 9 and 16; molecular marker (New England Biolabs, Cat. No. N3231L). Run on 2% agarose gel. Bands are annotated based on expected sizes for each amplicon

The need for candidates to be used in vaccine development call for in-depth genetic analysis of all possible candidates. In this light, many candidates with vaccine potential are studied for their suitability. Considering Ghana with unique malaria transmission dynamics, this study sought to shed light on the *eba-175* gene dimorphic forms circulating among patients. The study assessed spatio-temporal variation in the allelic dimorphism of EBA-175 (C-allele (714 bp) and F-allele (795 bp), Fig. 3) across different malaria transmission zones in 2019 and 2022.

Overall, F-allele was observed in higher frequency than C-allele, but a significant proportion of infections also carried mixed F/C alleles, which may be due to the multiplicity of infection in these high malaria transmission regions. In high transmission settings, mosquitoes are more likely to acquire and transmit a diverse array of parasite strains during blood meals, leading to co-infections in humans [29, 30].

Potential limitations of this study include the reliance on archived dried spot samples, which might have affected the integrity and quality of the extracted gDNA and may explain some of the failed assays. Multiple studies, however, have demonstrated the stability and quality of DNA extracted from properly stored DBS, even after extended periods [31, 32]. Also, sampling from selected health facilities could potentially introduce local sampling bias, however, the goal was to have representation across the three ecological zones, which should minimize any potential such bias [33]. Furthermore, the seasonality analysis should be interpreted with caution as climate change effects might cause different shifts in transmission between zones that were not accounted for.

In general, the data in current study agree well with reports from previous studies in Ghana, neighbouring Burkina Faso, Gambia, Nigeria, Gabon, and Equatorial Guinea [15, 17, 22, 34, 35], which also confirm the high prevalence of the F-allele across sub-Saharan Africa. The *eba-175* allelic distribution amongst the Ghanaian population highlights the genetic makeup of *P. falciparum*, with multiple alleles co-circulating within the same geographic area. The spatial and temporal prevalence of the F-allele across the population screened with eco-epidemiological variation suggests that the parasite population is largely panmictic with significant gene flow across time and space, which can be important for public health strategies. Thus, the high proportion of infections which carried CAMP/FCR-3 alleles further underscores the significant levels of transmission intensity resulting in high outcrossing in the mosquito midgut. Previous studies have also suggested immunological selection [15], and human demography and/or genetic background as key drivers of *eba-175* allelic dimorphism [36, 37]. The high prevalence of F-allele in African populations and high prevalence of C-allele in Latin American populations such as Brazil [15] suggests that malaria transmission, type of vectors and host factors may be structuring the diversity and distribution of these alleles.

This study did not observe any clear trend of rapidly decreasing frequency of F-allele with age, even though its frequency was highest among children under 5 years (Fig. 2). The behaviour of the F-allele from this study is consistent with data from Uganda where the F-allele was found to be predominant among children below 5 years [23]. The frequency of C-allele however generally increased with age across all age groups and is likely to be under much stronger immune selection than the F-allele [15, 21].

The predominance of the F-allele of across different ecological zones and time points suggests that any vaccine or therapeutic approach that effectively targets this antigen would have a broader success. However, the presence of a significant proportion of C-allele and mixed infections highlights the need for multivalent vaccine (or drug) formulations that include both F and C alleles of EBA-175 to ensure broad coverage against circulating parasite strains for higher public health impact. Furthermore, the persistence of mixed infections highlights the complexity of *P. falciparum* populations in high transmission settings, which may require more sophisticated intervention strategies to effectively reduce the parasite reservoir.

Malaria transmission in northern Ghana remains unimodal with the highest transmission season occurring during the rainy season. These transmission seasons however did not have any significant effect on the prevalence of the *eba-175* alleles. Thus, the observed pattern of dominance of the F-allele in clinical infections may be reflective of the malaria reservoir of asymptomatic infections that continues to fuel transmission during the low season, further studies need to confirm this or otherwise. The lack of seasonal variation in allele prevalence suggests that a successful PfEBA-175 based vaccine may have a broader year-round effect across infections with potential to achieve a greater public health benefit in Ghana and other malaria endemic settings where malaria transmission is seasonal.

The Geometric mean parasite density (GMPD) of children, 0 to 5 years showed higher parasite density compared to adults from 36 years and above (Table 1). The high GMPD infer increased susceptibility which can be attributed to their developing immune systems which may not effectively respond to the diverse array of *P. falciparum* genotypes. This highlights how children continue to stand a risk of high parasite density infection and severe malaria and the need for intensified protection measures for this vulnerable group, such as improved bed net coverage including the deployment of dual active long-lasting insecticidal nets to that are more effective against resistant mosquitoes and ensuring more children received all four rounds of seasonal malaria chemoprevention treatment during the high malaria season [38, 39]. Interestingly, the GMPD for mixed (F/C) infections was highest. This could be due to synergistic effects where different strains complement each other’s growth dynamics or evade the host's immune response more effectively; this was followed by the C-allele and the lowest was the F-allele. The results suggest that parasites carrying the F-allele, though common, may be slow growers as compared to strains carrying the C-allele.

## Conclusion

The study found the F-allele to be the most predominant form across all age groups, followed by the C-allele and mixed F/C alleles. No significant difference in allele prevalence was observed between the high malaria season (wet) and low malaria season (dry). Furthermore, no pattern was found in the distribution of geometric mean parasite densities between the different allelic forms, with the highest densities observed in mixed allele infections. The current study highlights the co-circulation of alleles in these high malaria endemic areas in Ghana.

## Data Availability

No datasets were generated or analysed during the current study.

## References

[1] Zekar L, Sharman T. *Plasmodium falciparum* malaria. StatPearls (Internet); Treasure Island (FL) StatPearls Publishing. 2024. 32310422

[2] WHO. World malaria report 2023. Geneva, World Health Organization; 2023.

[3] Cowman AF, Tonkin CJ, Tham WH, Duraisingh MT. The molecular basis of erythrocyte invasion by malaria parasites. Cell Host Microbe. 2017;22:232–45. 28799908 10.1016/j.chom.2017.07.003 PMC12801281

[4] Orlandi PA, Klotz FW, Haynes JD. A malaria invasion receptor, the 175-kilodalton erythrocyte binding antigen of *Plasmodium falciparum* recognizes the terminal Neu5Ac (alpha 2–3) Gal-sequences of glycophorin A. J Cell Biol. 1992;116:901–9. 1310320 10.1083/jcb.116.4.901 PMC2289329

[5] Tolia NH, Enemark EJ, Sim BKL, Joshua-Tor L. Structural basis for the EBA-175 erythrocyte invasion pathway of the malaria parasite *Plasmodium falciparum*. Cell. 2005;122:183–93. 16051144 10.1016/j.cell.2005.05.033

[6] Michon P, Stevens JR, Kaneko O, Adams JH. Evolutionary relationships of conserved cysteine-rich motifs in adhesive molecules of malaria parasites. Mol Biol Evol. 2002;19:1128–42. 12082132 10.1093/oxfordjournals.molbev.a004171

[7] Camus D, Hadley TJ. A *Plasmodium falciparum* antigen that binds to host erythrocytes and merozoites. Science. 1985;230:553–6. 3901257 10.1126/science.3901257

[8] Chen E, Paing MM, Salinas N, Sim BKL, Tolia NH. Structural and functional basis for inhibition of erythrocyte invasion by antibodies that target *Plasmodium falciparum* EBA-175. PLoS Pathog. 2013;9: e1003390. 23717209 10.1371/journal.ppat.1003390 PMC3662668

[9] Moll K, Chene A, Ribacke U, Kaneko O, Nilsson S, Winter G, et al. A novel DBL-domain of the *P. falciparum* 332 molecule possibly involved in erythrocyte adhesion. PLoS One. 2007;2:e477. 10.1371/journal.pone.0000477 PMC1868959 17534427

[10] Patarroyo ME, Alba MP, Rojas-Luna R, Bermudez A, Aza-Conde J. Functionally relevant proteins in *Plasmodium falciparum* host cell invasion. Immunotherapy. 2017;9:131–55. 28128713 10.2217/imt-2016-0091

[11] Jaskiewicz E, Jodłowska M, Kaczmarek R, Zerka A. Erythrocyte glycophorins as receptors for *Plasmodium* merozoites. Parasit Vectors. 2019;12:317. 31234897 10.1186/s13071-019-3575-8 PMC6591965

[12] Chowdhury P, Ray S, Chakraborty A, Sen S, Dasgupta AK, Sengupta S. Non-synonymous amino acid alterations in PfEBA-175 modulate the merozoite ligand’s ability to interact with host’s glycophorin A receptor. Infect Genet Evol. 2020;85: 104418. 32561295 10.1016/j.meegid.2020.104418

[13] Dolan SA, Proctor JL, Alling DW, Okubo Y, Wellems TE, Miller LH. Glycophorin B as an EBA-175 independent *Plasmodium falciparum* receptor of human erythrocytes. Mol Biochem Parasitol. 1994;64:55–63. 8078523 10.1016/0166-6851(94)90134-1

[14] Blair PL, Kappe SH, Maciel JE, Balu B, Adams JH. *Plasmodium falciparum* MAEBL is a unique member of the ebl family. Mol Biochem Parasitol. 2002;122:35–44. 12076768 10.1016/s0166-6851(02)00067-1

[15] Touré FS, Mavoungou E, Me Ndong JM, Tshipamba P, Deloron P. Erythrocyte binding antigen (EBA-175) of *Plasmodium falciparum*: improved genotype determination by nested polymerase chain reaction. Trop Med Int Health. 2001;6:767–9. 11679124 10.1046/j.1365-3156.2001.00789.x

[16] Cramer JP, Mockenhaupt FP, Möhl I, Dittrich S, Dietz E, Otchwemah RN, et al. Allelic dimorphism of the erythrocyte binding antigen-175 (eba-175) gene of *Plasmodium falciparum* and severe malaria: significant association of the C-segment with fatal outcome in Ghanaian children. Malar J. 2004;3:11. 15140262 10.1186/1475-2875-3-11 PMC420250

[17] Dittrich S, Schwöbel B, Jordan S, Vanisaveth V, Rattanaxay P, Christophel EM, et al. Distribution of the two forms of *Plasmodium falciparum* erythrocyte binding antigen-175 (eba-175) gene in Lao PDR. Malar J. 2003;2:23. 12901736 10.1186/1475-2875-2-23 PMC169188

[18] Perce-da-Silva DS, Banic DM, Lima-Junior JC, Santos F, Daniel-Ribeiro CT, de Oliveira-Ferreira J, et al. Evaluation of allelic forms of the erythrocyte binding antigen 175 (EBA-175) in *Plasmodium falciparum* field isolates from Brazilian endemic area. Malar J. 2011;10:146. 21615944 10.1186/1475-2875-10-146 PMC3138422

[19] Yang PK, Liang XY, Lin M, Chen JT, Huang HY, Lin LY, et al. Population genetic analysis of the *Plasmodium falciparum* erythrocyte binding antigen-175 (EBA-175) gene in Equatorial Guinea. Malar J. 2021;20:374. 34538247 10.1186/s12936-021-03904-x PMC8451130

[20] Sato S. *Plasmodium* - a brief introduction to the parasites causing human malaria and their basic biology. J Physiol Anthropol. 2021;40:1. 33413683 10.1186/s40101-020-00251-9 PMC7792015

[21] Adam AA, Amine AA, Hassan DA, Omer WH, Nour BY, Jebakumar AZ, et al. Distribution of erythrocyte binding antigen 175 (EBA-175) gene dimorphic alleles in *Plasmodium falciparum* field isolates from Sudan. BMC Infect Dis. 2013;13:469. 24103447 10.1186/1471-2334-13-469 PMC3851876

[22] Binks RH, Baum J, Oduola AM, Arnot DE, Babiker HA, Kremsner PG, et al. Population genetic analysis of the *Plasmodium falciparum* erythrocyte binding antigen-175 (eba-175) gene. Mol Biochem Parasitol. 2001;114:63–70. 11356514 10.1016/s0166-6851(01)00240-7

[23] Agwang C, Erume J, Okech B, Olobo J, Egwang TG. Age-dependent carriage of alleles and haplotypes of *Plasmodium falciparum* sera5, eba-175, and csp in a region of intense malaria transmission in Uganda. Malar J. 2020;19:361. 33032613 10.1186/s12936-020-03432-0 PMC7543040

[24] Osarfo J, Ampofo GD, Tagbor H. Trends of malaria infection in pregnancy in Ghana over the past two decades: a review. Malar J. 2022;21:3. 34983534 10.1186/s12936-021-04031-3 PMC8725495

[25] Forson AO, Hinne IA, Dhikrullahi SB, Sraku IK, Mohammed AR, Attah SK, et al. The resting behavior of malaria vectors in different ecological zones of Ghana and its implications for vector control. Parasit Vectors. 2022;15:246. 35804461 10.1186/s13071-022-05355-y PMC9270803

[26] WHO. Preparation of blood spots on filter paper. World Health Organization; 2016.

[27] WHO. Malaria microscopy quality assurance manual-version 2. Geneva, World Health Organization; 2016.

[28] WHO, UNICEF. Microscopy for the detection, identification and quantification of malaria parasites on stained thick and thin blood films in research settings (version 1.0): procedure: methods manual. Geneva, World Health Organization; 2015.

[29] Zhan Q, Tiedje KE, Day KP, Pascual M. From multiplicity of infection to force of infection for sparsely sampled *Plasmodium falciparum* populations at high transmission. medRxiv. 2024:2024.02.12.24302148 (pre-print). 10.7554/eLife.100076 PMC13290237 42333925

[30] Touray AO, Mobegi VA, Wamunyokoli F, Herren JK. Diversity and Multiplicity of *P. falciparum* infections among asymptomatic school children in Mbita, Western Kenya. Sci Rep. 2020;10:5924. 10.1038/s41598-020-62819-w PMC7125209 32246127

[31] Nain M, Sinha A, Sharma A. Dried blood spots: a robust tool for malaria surveillance in countries targeting elimination. J Vector Borne Dis. 2023;60:11–7. 37026215

[32] Choi EH, Lee SK, Ihm C, Sohn YH. Rapid DNA extraction from dried blood spots on filter paper: potential applications in biobanking. Osong Public Health Res Perspect. 2014;5:351–7. 25562044 10.1016/j.phrp.2014.09.005 PMC4281615

[33] Matrevi SA, Adams T, Tandoh KZ, Opoku-Agyeman P, Bruku S, Ennuson NA, et al. Putative molecular markers of *Plasmodium falciparum* resistance to antimalarial drugs in malaria parasites from Ghana. Front Epidemiol. 2024;4:1279835. 38456076 10.3389/fepid.2024.1279835 PMC10910922

[34] Soulama I, Bougouma EC, Diarra A, Nebie I, Sirima SB. Low-high season variation in *Plasmodium falciparum* erythrocyte binding antigen 175 (eba-175) allelic forms in malaria endemic area of Burkina Faso. Trop Med Int Health. 2010;15:51–9. 19891760 10.1111/j.1365-3156.2009.02415.x PMC2858779

[35] Touré FS, Bisseye C, Mavoungou E. Imbalanced distribution of Plasmodium falciparum EBA-175 genotypes related to clinical status in children from Bakoumba. Gabon Clin Med Res. 2006;4:7–11. 16595788 10.3121/cmr.4.1.7 PMC1435655

[36] Ndiaye M, Faye B, Tine R, Ndiaye JL, Sylla K. Genetic analysis of erythrocyte binding antigen 175 (EBA-175), apical membrane antigen (AMA-1) and merozoite surface protein 3 (MSP-3) allelic types in *Plasmodium falciparum* isolates from rural area in Senegal. Malar Chemother Control Elimin. 2014;3:113.

[37] Soulama I, Bigoga JD, Ndiaye M, Bougouma EC, Quagraine J, Casimiro PN, et al. Genetic diversity of polymorphic vaccine candidate antigens (apical membrane antigen-1, merozoite surface protein-3, and erythrocyte binding antigen-175) in *Plasmodium falciparum* isolates from western and central Africa. Am J Trop Med Hyg. 2011;84:276. 21292899 10.4269/ajtmh.2011.10-0365 PMC3029182

[38] Piccaluga PP, Rodriguez-Morales AJ. Malaria: recent advances and new perspectives. IntechOpen, 2023.

[39] Bandolo FMN. Heavily indebted poor countries (HIPC) initiative in cameroon and the fight to reduce malaria related under-five mortality. Master Thesis. Univ ersity of Akershus, Oslo. 2012.

